# Iodine Status and Thyroid Function in a Group of Seaweed Consumers in Norway

**DOI:** 10.3390/nu12113483

**Published:** 2020-11-13

**Authors:** Inger Aakre, Lidunn Tveito Evensen, Marian Kjellevold, Lisbeth Dahl, Sigrun Henjum, Jan Alexander, Lise Madsen, Maria Wik Markhus

**Affiliations:** 1 Department of Seafood and Nutrition, Institute of Marine Research, NO-5817 Bergen, Norway; lidunnt@hotmail.com (L.T.E.); lisbeth.dahl@hi.no (L.D.); lise.madsen@hi.no (L.M.); maria.wik.markhus@hi.no (M.W.M.); 2 Department of Clinical Science, Faculty of Medicine, University of Bergen, NO-5020 Bergen, Norway; 3 Department of Contaminants and Biohazards, Institute of Marine Research, NO-5817 Bergen, Norway; Marian.kjellelvold@hi.no; 4 Department of Nursing and Health Promotion, Oslo Metropolitan University (OsloMet), NO-0130 Oslo, Norway; shenjum@oslomet.no; 5 Division of Infection Control, Environment and Health, Norwegian Institute of Public Health, NO-0213 Oslo, Norway; Jan.Alexander@fhi.no; 6 Department of Biology, University of Copenhagen, DK-2100 Copenhagen Ø, Denmark

**Keywords:** iodine, seaweed, urinary iodine status, thyroid function, iodine excess, food and nutrition security, new marine resources

## Abstract

Seaweeds, or macroalgae, may be a good dietary iodine source but also a source of excessive iodine intake. The main aim in this study was to describe the iodine status and thyroid function in a group of macroalgae consumers. Two urine samples were collected from each participant (*n* = 44) to measure urinary iodine concentration (UIC) after habitual consumption of seaweed. Serum thyroid stimulating hormone (TSH), free thyroxine (fT_4_), free triiodothyronine (fT_3_), and peroxidase autoantibody (TPOAb), were measured in a subgroup (*n* = 19). A food frequency questionnaire and an iodine-specific 24 h recall were used to assess iodine intake and macroalgae consumption. The median (p25–p75) UIC was 1200 (370–2850) μg/L. Median (p25–p75) estimated dietary iodine intake, excluding macroalgae, was 110 (78–680) μg/day, indicating that seaweed was the major contributor to the iodine intake. TSH levels were within the reference values, but higher than in other comparable population groups. One third of the participants used seaweeds daily, and sugar kelp, winged kelp, dulse and laver were the most common species. Labelling of iodine content was lacking for a large share of the products consumed. This study found excessive iodine status in macroalgae consumers after intake of dietary seaweeds. Including macroalgae in the diet may give excessive iodine exposure, and consumers should be made aware of the risk associated with inclusion of macroalgae in their diet.

## 1. Introduction

Iodine is an essential micronutrient required for the synthesis of thyroid hormones [1]. It is found in few foods of terrestrial origin [2], however, in countries where fodder is enriched with iodine, milk, dairy products and eggs may be good dietary sources [3,4]. Marine foods, such as fish and shellfish are naturally high in iodine due to the high iodine concentration in seawater [2]. Seaweeds, or macroalgae, may contain high amounts of iodine [5,6]. Macroalgae is commonly used in Asian cultures [7,8,9], especially Japan, China and Korea, where edible macroalgae are commonly consumed in soups, sushi, as seaweed salads, snacks and in other dishes [7,9,10,11]. During recent years, macroalgae has entered the global food market, and has become increasingly popular in the western part of the world [12,13]. In Europe, macroalgae represents a potential new dietary iodine source, and has also been considered as an especially healthy food or even “super food” [14,15,16,17].

Both iodine deficiency and excess may increase the risk of developing a thyroid disorder, hence, the relationship between iodine intake and the risk of developing a thyroid disorder is U-shaped [18]. Excessive iodine intake may develop into hypothyroidism or hyperthyroidism, both with or without goiter, depending on the initial and current iodine status and current thyroid function [19,20]. Additionally, acute excessive iodine intake may adversely impact the thyroid [21]. This may be of particular concern for vulnerable groups, such as pregnant women and individuals with thyroid autoimmunity [19,20]. Excessive iodine intake from dietary macroalgae has been associated with thyroid dysfunction (subclinical hypothyroidism) in otherwise healthy adults in Asian populations [22,23].

Although consumption of macroalgae has become increasingly popular in western countries during recent years, little is known about the iodine status and thyroid function in macroalgae consumers. The European Food Safety Authorities (EFSA) has requested more knowledge on iodine content of dietary seaweed as well as data on macroalgae consumption [24]. Further, the Norwegian Committee for Food and Environment has highlighted consumers of macroalgae as a potential risk group for excessive iodine intakes in the Norwegian population [25]. The aim of this study is to describe iodine intake and iodine status in a group of macroalgae consumers in Norway, and to report on parameters of thyroid function. We also describe the frequency of habitual macroalgae consumption, where the dietary macroalgae are procured, and the species that are consumed.

## 2. Materials and Methods

### 2.1. Recruitment of Study Group

Convenience sampling was used to recruit 44 consumers of macroalgae during September, October and November 2019. We included frequent macroalgae consumers, defined as habitual consumption of macroalgae on a weekly basis. Information about the study and an invitation to participate were broadcast on platforms where consumers of macroalgae were likely to retrieve the information, such as webpages and social media groups. This was determined by searching the web and by talking to key informants from different macroalgae social networks of Norway. We also contacted stores in Oslo and Bergen, the two largest cities in Norway, where macroalgae can be purchased, i.e., health food stores, international food stores and the fish market in Bergen. When participants were enrolled, they were encouraged to invite acquaintances to participate in order to facilitate snowball sampling [26]. Participants provided informed written consent. The present study was conducted according to the guidelines in the Declaration of Helsinki and was approved by the Regional Committee for Medical and Health Research Ethics Norway (2015/1845).

### 2.2. Sample

**Figure 1:**
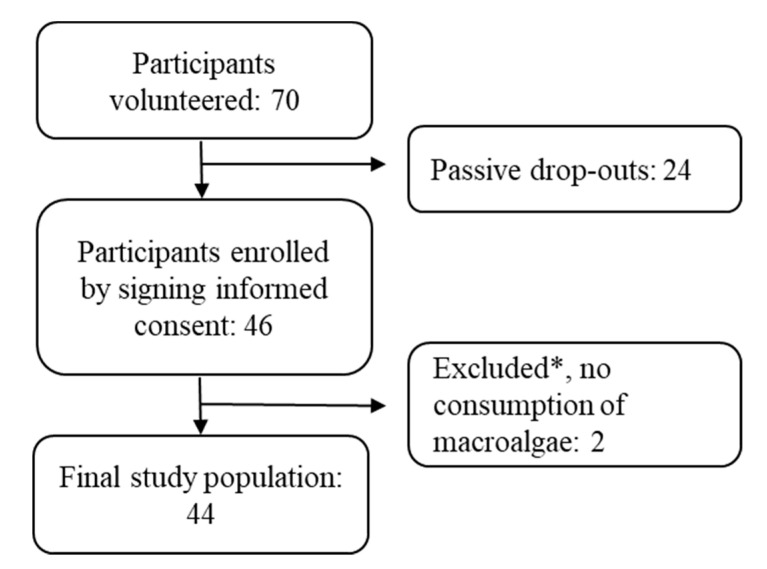
Participation flow. * Two participants were excluded as they used freshwater algae and an algae supplement for omega-3 fatty acids.

In total, 70 individuals volunteered for the study. Of these, 24 individuals chose not to participate for unknown reasons, leaving 46 participants enrolled by signing and returning the written informed consent form. Two individuals not consuming seawater macroalgae were excluded from the analysis, leaving a study population of 44 participants. Participation flow is found in Figure 1.

The participants (*n* = 44) answered questions on background information (age, weight, sex, height, educational level, smoking habits, country of birth, years living in Norway, languages, known thyroid disease, use of thyroid medication and planning of pregnancy). Two participants reported use of Levaxin. We do not have data regarding use of other medications than thyroid medication.

### 2.3. Urinary Iodine Concentration

All participants delivered two urine samples. The participants were encouraged to consume macroalgae in the same manner as usual, and were instructed to sample their urine 3–4 and 7–8 h after consumption. The pre-determined sampling intervals were chosen based on the investigation of urinary iodine excretion in humans after consumption of *Ascophyllum nodosum*, conducted by Combet et al. [27]. Urine samples were stored refrigerated (0–4 °C) until delivery at the Institute of Marine Research (IMR), where the samples were stored at minus 20 °C pending analysis of urinary iodine concentration (UIC), by inductively coupled plasma mass spectrometry (ICP-MS). For the determination of iodine, 500 µL urine was diluted in 4.5 mL 1% tetramethylammonium hydroxide (TMAH) and filtered using a sterile membrane with a 0.45 µm pore size and a single-use syringe. Samples were analyzed against a urine calibration curve (standard addition curve). Internal validity of the method was verified with certified reference material (SRM); Seronorm Trace Elements Urine. The measurement uncertainty for iodine is 20% for the whole measurement range [28].

The two urine samples for each individual were used to calculate the mean UIC for each participant, and further the median UIC for the group. Daily iodine intake was estimated from UIC using the following equation: daily iodine intake = UIC (µg/L) ÷ 0.92 × (0.0009 L/hour/kg × 24 h/day) × body weight (kg) [29].

We assessed whether UIC had remained stable, increased or decreased between the first and second urine sample. Based on a measurement uncertainty of 20%, a change larger than ±10% from the first urine sample to the second urine sample was considered as an increase or decrease.

### 2.4. Thyroid Hormones in Blood Samples

Blood samples for analysis of thyroid function were obtained from a subgroup (n = 19). For logistical reasons, blood samples were not taken from participants (n = 25) living far from where the clinical laboratory (Fürst Medical Laboratories) was located (Oslo and Bergen). Venous blood samples for serum preparation were collected in BD Vacutainer^®^ SST™ vials II Advanced, and set to coagulate for a minimum of 30 min. Within 60 min after phlebotomy, the samples were centrifuged (1000–3000 G, room temperature, 10 min). Post-separation, serum samples were sent to Oslo and stored refrigerated (2–8 °C) overnight, pending analysis at Fürst Medical Laboratories in Oslo, Norway, the following day. Thyroid stimulating hormone (TSH), free thyroxine (fT_4_), free triiodothyronine (fT_3_) and thyroid peroxidase antibodies (anti-TPO) were analyzed in serum using magnetic separation and detection by chemiluminescence, labeled with acridinium ester, on an Advia Centaur XPT Immunoassay system (Siemens Healthcare Diagnostics Inc., Tarrytown, NY, USA). Reference values established by Fürst Medical Laboratories were used.

### 2.5. Assessment of Iodine from Food and Dietary Supplements, Excluding Macroalgae

Iodine intake from iodine-rich foods and iodine-containing supplements was calculated for the last 24 h (I24-h), and habitual iodine intake was calculated from a short food frequency questionnaire (FFQ). The I24-h included questions about intake of milk and yogurt (number of glasses), cheese, eggs (including dishes), and fish (lean and fatty fish for dinner and/or as spread), as these are the main food items contributing to iodine intake in Norway. Milk and dairy products are together with egg and fish the most important dietary iodine sources in Norway [30] since the cow’s fodder is enriched with iodine. Use of iodized salt by the food industry is not mandatory in Norway, and as the available iodized table salt is only allowed to contain up to 5 µg iodine/g salt, the contribution is neglectable. The reported food intakes were multiplied with the iodine concentration for each specified food to obtain the estimated iodine intake. The FFQ included 32 different food items, with frequencies ranging from “*rarely/never*” to “*five times daily or more*”. The recall period was the last four weeks. Of the 32 food items, 11 questions assessed iodine-rich foods, including five questions about milk and dairy, five questions about fish and fish products and one question about eggs. Reported intake of milk, cheese, fish, and egg were converted to daily amounts and multiplied with the iodine concentration for each food item. Standard portion sizes for each of the different food items were used [31]. In all calculations, iodine concentrations reported in the Norwegian Food Composition Table [32] or in Nerhus et al. [33] were used. We applied values from Nerhus et al. for lean fish, cheese, whey cheese and eggs. To account for iodine contributed by foods and dishes not covered by the assessed food items, 30 µg was added to each estimated total intake as suggested by others [34].

Participants were asked to report if they used iodine-containing supplements (non-algae), such as multivitamins or mineral supplements, and report the quantity of iodine and frequency of consumption. The daily quantity of iodine contributed by iodine-containing supplements was calculated and added to both the calculated 24 h intake (I24-h) and the habitual intake from food (FFQ).

The participants were asked about vegetarian practices, where “vegetarians” were defined by exclusion of meat and fish and inclusion of milk, dairy products and eggs. We defined those excluding meat, but including fish, milk and eggs as “pesco-vegetarians” and those excluding meat and milk but included fish and eggs as “pesco-vegetarians excluding milk”. “Vegans” were defined by exclusion of all animal source products in the diet. The variable vegetarian practice (n = 7) included all vegetarian/vegan practices combined.

### 2.6. Assessment of Macroalgae Consumption

Questions regarding intake of macroalgae in the diet were divided into three categories; (1) whole food macroalgae, (2) macroalgae-containing foods and (3) macroalgae-containing supplements. Macroalgae in the diet were investigated as habitual consumption (FFQ) using the following frequency intervals: “*daily*”, “*4–6 times/week*”, “*1–3 times/week*”, “*less than once a week*” and “*other*”, and as consumption in the last 24 h. Further, questions regarding types of products, macroalgae species, where the product was purchased, and how it was used as a part of the diet were included. All questions regarding macroalgae in the diet were open ended except for given frequency intervals. In addition, the participants provided detailed information about macroalgae consumed on the same day as they took the urine samples.

### 2.7. Assessment of Iodine from Macroalgae

There are no established portion sizes for edible macroalgae. Therefore, only participants who provided sufficient information about the quantity of macroalgae intake and the iodine concentration from the package labelling could be included for the estimation of iodine intake from macroalgae. This information was only provided by a small number of participants (*n* = 10 for the 24 h intake, *n* = 11 for the habitual intake). Therefore, the difference between the estimated iodine intake from UIC and the iodine intake from food and non-algae supplements estimated from the survey was used to estimate iodine intake from macroalgae for all participants (*n* = 44), using the following calculation: estimated iodine intake from macroalgae = estimated iodine intake from UIC - estimated iodine intake from food (I-24h) µg/day. For example: estimated iodine intake from UIC = 3000 µg/day; estimated iodine intake from food (I-24h) = 200 µg/day. Estimated iodine intake from macroalgae: 3000 − 200 µg/day = 2800 µg/day. For three (I-24H) and five (FFQ) participants, the values were negative, as the estimated iodine intakes from food were probably overestimated. In those cases, the value zero was imputed into the dataset and used when calculating central and variation values of the variable. 

### 2.8. Statistical Methods

IBM SPSS 26 (Statistical Package for the Social Sciences) was used to perform all statistical analyses. *p*-value < 0.05 was used as statistical value of significance in all tests, unless other is noted. Dependent variables were checked for normality using Q–Q plots and the Shapiro–Wilk test. Due to the skewed distribution and low number, non-parametric tests were used. Independent group differences were examined using the Mann–Whitney U test for comparison of two groups, and Kruskal–Wallis test was used for comparison of categories with more than two groups. Spearman’s rank correlation was used to evaluate the linear relationship between continuous variables.

## 3. Results

Characteristics of the study participants are presented in Table 1. The mean age was 46 years, and 61% were women. Seventy-five percent were born in Norway, while 23% were born in other European countries and one person outside Europe. The participants born outside Norway had significantly higher median UIC (2225 µg/L) than those born in Norway (620 µg/L), *p* = 0.01. The 16% who reported a vegetarian dietary practice had significantly lower median UIC (165 ug/L) than those not reporting a vegetarian diet (1285 ug/L), *p* = 0.045.

UIC measured 3–4 and 7–8 h after ingestion of macroalgae is presented in Table 2, together with habitual and 24 h iodine intake. Median (p25–p75) UIC for the first and second urine sample was 1080 (300–2150) µg/L and 1050 (340–3450) µg/L, respectively. The median daily iodine intake estimated from UIC, representing total iodine intake from all sources, was 2430 µg/day. The estimated median iodine intake from non-algal foods and supplements was 110 µg/day for the last 24 h, while the habitual iodine intake (FFQ) was 260 µg/day. The estimated iodine intake from macroalgae alone, based on the estimated iodine intake from UIC, was 2160 µg/day and 2200 µg/day for the habitual (FFQ) and 24 h intake, respectively.

As shown in Table 2, the median UIC was similar between the first and the second sample. The mean SD UIC was considerably higher for the second sample; however, the differences were not statistically significant (*p* = 0.717). Stability in UIC from the first to the second urine sample is shown in Table S1. There was an increase in UIC from the first to the second sample for 48% (*n* = 21) of the participants, while a decrease for 32% (*n* = 14) and 21% (*n* = 9) remained stable. There was a significant correlation between UIC from the two time-points. A visual presentation of the change in UIC is presented in Figure S1a–c.

The participants reported the type of macroalgae consumed on the day of the urine sample. The median UICs were highest among the participants who reported consumption of sugar kelp, with a median UIC of 2900 µg/L (Table 3).

Descriptive data of thyroid hormone levels for the subsample (*n* = 19) are presented in Table 4. Of the 19 participants in the subgroup, one participant had a TSH value above the laboratory reference (4.0 mIU/L), and two participants were peroxidase autoantibody (TPOAb) positive. The median (p25–p75) UIC (mean value of the first and the second sample) in the subsample was 360 (145–1150) µg/L. Of note, these participants had significantly lower median UIC than the participants who did not deliver a blood sample (median: 1800 µg/L), *p* < 0.005.

Table 5 shows the frequency of habitual seaweed consumption (last four weeks) and procurement details. Most participants used whole macroalgae products (*n* = 41). Products with macroalgae as an ingredient were used by 35 of the participants, and macroalgae as a supplement by five participants. Most of the participants used products from more than one category. In the category of whole food products, 23% consumed macroalgae daily. Across the product categories, about 30% reported daily consumption of macroalgae. For the wholefood macroalgae, 42% were personally harvested by the participants, and 42% were store-bought products. About 29% of the participants consuming foods with macroalgae had bought the products in a chain store. A total of 22% of the whole food products had iodine declaration on the package, whereas 14% and 40% of foods and supplements, respectively, had iodine declaration. The participants reported that the declared content of iodine ranged from 63 µg/g to 35,000 µg/g for whole food macroalgae and 0.6 µg/g to 3360 µg/g for macroalgae containing foods. Sugar kelp (*Saccharina latissima*), winged kelp (*Alaria esculenta*), and dulse (*Palmaria palmata*) were the species that most participants reported to consume habitually in the category of whole macroalgae products. An overview of macroalgae species consumed habitually (FFQ) is reported in Table 6.

Several reasons for including macroalgae in the diet were given by the participants (Table S2. The most common reasons were taste (*n* = 27), richness in nutrients (*n* = 15) and because it was considered sustainable (*n* = 9). Other reasons given were positive health effects/healthy; exciting; to ensure iodine in the diet; a substitute for fish; healing effect; and to reduce salt intake. Macroalgae was used in different ways by the participants (Table S3, where seasoning/spice (*n* = 24), sushi (*n* = 8) and snacks (*n* = 8) were the most common ways.

## 4. Discussion

The median (p25–p75) UIC among the 44 macroalgae consumers included in this study was 1200 (370–2850) µg/L after macroalgae consumption, and the estimated median (p25–p75) iodine intake from macroalgae alone was 2200 (280–4060) µg/day. Sugar kelp, winged kelp, dulse and laver were the most used macroalgae species. The participants who had included sugar kelp in their diet the day of the urine sampling had a median UIC of 2900 µg/L, which was higher than for the other reported species. Thirty percent reported habitual daily use of macroalgae, while 23% used macroalgae 4–6 times/week and about 50% included macroalgae in the diet 1–3 times/week or less frequently.

The median UIC considerably exceeds the cut-off value of 300 μg/L from WHO/UNICEF/IGN, indicating excessive iodine nutrition [35]. Further, the median estimated iodine intake of 2430 μg/day was four times higher than the tolerable upper intake level (UL) from EFSA of 600 μg/day [36]. Three-quarters of the participants exceeded the UL of 600 μg/day, while 64% exceeded the UL from The National Academy of Medicine (NAM), formerly called the Institute of Medicine, of 1100 μg/day [29]. Fifty-five percent exceeded the lowest observed adverse effect level (LOAEL) for iodine intake of 1800 μg/day, used both by EFSA and NAM. Therefore, the risk of adverse health effects from excessive iodine intake in this study population can be considered high. However, a large inter-individual variation in UIC was seen, as the measured UIC ranged from 70–16,000 μg/L. We believe this indicates large differences in iodine concentration in different macroalgae species, and/or large differences in the quantity of macroalgae consumed. This is in line with an Irish study, examining iodine content of 19 commonly consumed macroalgae species, where the iodine content between species was highly variable [37]. For the species with the highest iodine content, sugar kelp, only 0.04 g was required to meet the daily recommended daily intake of 150 µg [37]. 

The median daily iodine intake from food alone was considerably lower than the UIC. This indicates that macroalgae were the main contributor to dietary iodine in this study. Due to the design of the study and the lack of accurate data on the iodine content of the consumed macroalgae products, we cannot report on the bioavailability of iodine. However, as the contribution of iodine from macroalgae was high, and by far exceeded the contribution from other foods, the absorbed amount of iodine is likely to be substantial. Bioavailability of iodine from macroalgae in humans is estimated to vary from 50 to 100% [22,38,39]. The difference in bioavailability between species has been suggested to be related to the share of iodine stored in inorganic and organic form, such as mono- and di-iodothyrosine (MIT and DIT), in macroalgae, where humans absorb inorganic iodine, such as potassium iodine (KI) or iodate (IO_3_-), more effectively than organically bound iodine, e.g., MIT and DIT [38]. However, factors such as iodine status and current thyroid function may also be of importance for iodine uptake in humans [36].

In this study, one participant had subclinical hypothyroidism, as indicated by a TSH above the reference value, and two had positive TPOAb with thyroid function tests within the laboratory references. Compared with median TSH levels for men and women (1.5 mIU/L) in a large-scale Norwegian study (Nord-Trøndelag Health Study (HUNT)) [40], the median TSH level of 1.8 mIU/L observed in the present study was higher. Similarly, the mean serum TSH (2.0 ± 0.9 mIU/L) was higher than mean TSH (1.5 (±0.02) mIU/L) of a disease-free population in the U.S. National Health and Nutrition Examination Survey (NHANES III) [41]. In our study, we cannot say whether the TSH levels increased due to macroalgae ingestion because of the nature of the observational study design.

The participants providing blood samples (*n* = 19) had significantly lower median UIC (390 µg/L) than the remaining participants (*n* = 25, UIC: 1800 µg/L). Of note, the median estimated daily iodine intake from UIC for the subgroup was 540 µg/day and did not exceed the UL by EFSA of 600 µg/day, hence, one would not expect an impact on thyroid function. Thyroid function, however, might have been differently affected in the remaining group having an excessive iodine intake. Additionally, the time of the blood samples were not standardized according to macroalgae intake, thus, a transient rise in TSH may have occurred unnoticed. Further studies are required to examine thyroid function in macroalgae consumers with an excessive iodine intake. Previously, several case studies have reported iodine-induced thyroid dysfunction in adults consuming seaweed [42,43,44,45]. Thyroid dysfunction has also been seen in new-born babies after excessive maternal iodine intake from macroalgae [46,47]. A Japanese study reported elevated TSH after ingestion of high iodine from Kombu [22], and the authors advised against consuming excessive amounts of seaweed. Three population studies found an increase in UIC and TSH after macroalgae supplementation in euthyroid adults [8,27,48]. In all three studies, UIC and hormone levels returned to baseline after cessation of macroalgae supplementation. These studies were of short duration and do not reflect the effect that excessive iodine may have on the thyroid gland after years of macroalgae consumption. In a population with long-term excessive iodine exposure, the prevalence of subclinical and overt hypothyroidism was 14% and 4.5% in postpartum women [49], and 22% and 1.5% in a follow-up three years later [50]. The prevalence of subclinical hypothyroidism in young children from the same population was 9.3% [51]. In a systematic review and meta-analysis, the risk of subclinical hypothyroidism was significantly higher among men and women with iodine excess than among those with adequate iodine intakes [52]. Furthermore, the systematic review showed a dose-related increase in the prevalence of subclinical hypothyroidism as the level of UIC increased. Hence, macroalgae with a high concentration of iodine should be used with caution, especially in vulnerable groups such as pregnant women, as the fetus is susceptible for high maternal iodine intake and maternal thyroid function [53,54]. Further, as a large cohort study found that even mildly elevated TSH preconception could result in negative pregnancy outcomes, women in fertile age should also use high-iodine macroalgae with care [55]. Other known vulnerable groups to iodine excess are people with thyroid disease or thyroid autoimmunity [56].

Macroalgae were most commonly used as a seasoning-/spice-/flavor enhancer. Sugar kelp (*Saccharina latissima*) was the most popular species in our study. Concentrations of iodine in sugar kelp are reported as high as 7200 µg/g dry weight [57]. After sugar kelp, winged kelp (*Alaria esculenta*) and dulse (*Palmaria palmata*) were the species that most participants mentioned in the dietary survey in the category of whole macroalgae products. Winged kelp has been shown to be high in iodine (181–1070 µg/g dry weight), while dulse is lower (72–293 µg/g dry weight) [57].

A large share of the products the participants reported to consume were not labeled with iodine concentration. Similarly, in the UK, only 22 products out of 224, (10%), stated information regarding iodine content [12]. Due to variations in iodine concentration with season, harvesting location, age, and size of the macroalgae, storage and processing conditions, the iodine concentration is difficult to determine without specific analysis of products. The iodine concentration for the same species may vary considerably [5]. For the products in our study with declaration of iodine, the concentration was highly variable, ranging from 63 µg/g to 35,000 µg/g for whole food macroalgae and 0.6 µg/g to 3360 µg/g for macroalgae containing foods. With such variations in iodine content and with products without iodine declaration, it is difficult to decide if a product is safe to consume.

### Strengths and Limitations

This is the first study to examine iodine status and thyroid function among macroalgae consumers in Norway, thereby providing new insights into an area of limited research. The urine samples in this study were collected with a predetermined time-interval (3–4 h and 7–8 h) after consumption of macroalgae. These time intervals were chosen based on the investigation of urinary iodine excretion in humans after consumption of *Ascophyllum nodosum*, [27]. However, several different macroalgae species were used among the participants in this study, and the excretion curve may be different due to the varying bioavailability of iodine from different species and/or foods. It is considered a strength that we have two urine samples per participant, however, it must be noted that UIC from this study provides information about the iodine status after macroalgae consumption. If macroalgae are consumed only once a week, the total iodine intake per week may remain low, and the UIC is only representative for a day with consumption of macroalgae.

During the participant recruitment, there were 24 passive dropouts, meaning that they reported interest in the study and received a consent form and the necessary supplies to participate, but never followed up further. We do not know whether these individuals were different from the study population. Additionally, the sample size was small, and we cannot exclude the possibility of selection bias. The subsample providing information about thyroid function was small and not representative of the whole study population, as the median UIC differed significantly from the group that only provided urine samples. Conclusions about thyroid function for the whole study sample can therefore not be drawn.

Macroalgae was divided into three product categories: macroalgae as a whole food, products with macroalgae as an ingredient, and macroalgae as a supplement, such as pills/tablets or powder. Most participants were recruited from specific areas where harvested macroalgae were used as whole food. Due to limited time, participants using commercially available macroalgae products were probably underrepresented, as recruitment in health food stores, global food stores or chain stores was time-consuming.

Macroalgae may also be a source of contaminants, such as heavy metals and especially inorganic arsenic [58]. The potential exposure risk of these, and other undesirable components from macroalgae, was not evaluated in this study and requires further investigation.

## 5. Conclusions

Wholefood macroalgae was the most consumed macroalgae product, and sugar kelp, winged kelp, dulse and laver were the most common species. Iodine status in macroalgae consumers is excessive after intake of macroalgae. The UIC among the participants was highly variable, ranging from 70–16,000 µg/L, indicating a large variation in iodine concentrations in the macroalgae products consumed, confirmed by the reported iodine content of the declared products. However, for many products, iodine declaration was lacking. Macroalgae with low-level iodine used in appropriate amounts may be a good dietary source of iodine. Still, precaution must be taken before consuming macroalgae as it may give a high iodine exposure. Population groups vulnerable to high iodine intakes should avoid consuming macroalgae in order to reduce the risk of negative health consequences of excessive iodine intakes. Our study sample was small, and larger studies preferably with a controlled design including repeated measures, would be useful for future research within this topic. Studies examining the content and bioavailability of iodine and heavy metals in macroalgae and macroalgae products are also warranted.

## Figures and Tables

**Table 1:** Descriptive characteristics of seaweed consumers in Norway (*n* = 44) ^a^.

Characteristics ^d^		UIC ^b^ µg/L	*p*-Value ^c^
Age, mean	46.1 ± 12.4		
Male	16 [36.4]	1400 (428–2119)	1.00
Female	27 [61.4]	825 (250–5200)	
BMI, kg/m^2^	25.4 ± 5.4		
Country of birth			0.01
Norway	33 [75]	620 (226–2550)	
Other	11 [25]	2225 (1550–5700)	
Education level			
≤High school	10 [23.3]	935 (403–1725)	0.081
≤4 years university/college	10 [23.3]	402 (183–1676)	
>4 years university/college	23 [53.5]	2225 (655–5700)	
Smoking habits			
No	27 [61.4]	1250 (620–2950)	0.366 ^e^
Daily/Occasionally	4 [9.1]	2200 (496–11213)	
Former smoker	12 [27.3]	215 (135–1763)	
Smokeless tobacco (Snus)			
Daily	1 [2.3]		
Self-reported thyroid disease			
Hypothyroidism	3 [6.8]		
Number of children	1.3 ± 1.5		
Planned pregnancy, next two years	1 [2.3]		
Vegetarian practice	7 [15.9]	165 (85–1800)	0.045 ^f^
Vegetarian	1 [2.3]		
Vegan	2 [4.5]		
Pesco-vegetarian	2 [4.5]		
Pesco-vegetarian, excluding milk	2 [4.5]		
Use of iodine containing supplement	5 [11.4]		

^a^ Values are presented as mean ± SD, median (p25–p75), and n [%]. ^b^ Urinary iodine concentration (UIC) is given as the median value based upon a mean of two urine samples taken 3–4 and 7–8 h after seaweed consumption. ^c^ Differences between groups were tested using Mann–Whitney U or Kruskal–Wallis test. ^d^ One missing value for sex, education and smoking habits. ^e^ Tested between smokers/former smokers and non-smokers. ^f^ Tested between vegetarian practice (*n* = 7) and not vegetarian practice (*n* = 37).

**Table 2:** Urinary iodine concentration (UIC), habitual iodine intake from food and supplements ^a^, 24 h iodine intake from food and supplements ^a^ and estimated iodine intake from UIC (*n* = 44).

UIC, µg/L	Median	p25	p75	Mean ± SD	Min, Max	P ^f^
1st urine sample ^b^	1080	300	2150	1840 ± 2360	70, 12,000	0.171
2nd urine sample ^c^	1050	340	3450	2500 ± 3450	70, 16,000	
Mean 1st and 2nd urine sample	1200	370	2850	2170 ± 2770	80, 14,000	
**Iodine intake, µg/day**						
I-24H from food and non-algae supplements	110	70	970	390 ± 440	30, 1140	
FFQ from food and non-algae supplements	260	150	380	340 ± 490	30, 3300	
Daily iodine intake estimated from UIC ^d^	2430 ^g^	500	4630	3750 ± 4770	120, 25,660	
**Estimated iodine intake from seaweed only ^e^, µg/day**						
24 h intake (I-24H)	2160	280	4270	3450 ± 4720	0, 25,520	
Habitual intake (FFQ)	2200	280	4060	3420 ± 4750	0, 25,530	

^a^ Excluding iodine contribution from macroalgae ^b^ Approximately 3–4 h after intake of macroalgae. ^c^ Approximately 7–8 h after intake of macroalgae. ^d^ Estimated 24 h iodine intake from UIC = mean UIC × 0.0235 × body weight [28]. ^e^ Calculated from estimated iodine intake from UIC-estimated iodine intake from food and supplements. ^f^ Differences between 1st and 2nd urine sample tested with the Mann–Whitney U test. ^g^ A total of 75% (*n* = 33) had an estimated intake above 600 µg/day, 64% (*n* = 28) had an estimated intake above 1100 µg/day, and 55% (*n* = 24) had an estimated intake above 1800 µg/day. FFQ: Food frequency questionnaire.

**Table 3:** Reported macroalgae consumption on the day of the urine sampling and UIC (µg/L) among the participants within the reported species consumed (*n* = 44).

Macroalgae Type	Macroalgae Consumption the Day of Urine Sample	UIC, µg/L
	n [%]	Median (p25–p75)
**Sugar kelp**	10 [23]	2900 (1170–6587)
**Mix ^a^**	12 [27]	1350 (629–4613)
**Badderlocks, winged kelp**	9 [21]	1650 (565–1800)
**Rockweed, egg wrack**	4 [9]	335 (123–596)
**Oarweed**	1 [2]	-
**Wakame**	1 [2]	-
**Dulse**	1 [2]	-
**Laver**	1 [2]	-
**Unspecified**	5 [11]	250 (144–1305)

^a^ The species reported in the category of mix were dulse, wakame, sugar kelp, badderlocks, bladderwrack and horned wrack.

**Table 4:** Thyroid hormone levels (thyroid stimulating hormone (TSH), free triiodothyronine (fT_3_), and free thyroxine (fT_4_)) and thyroid peroxidase antibodies (anti-TPO) in seaweed consumers in Norway (*n* = 19).

Thyroid Hormones and TPOAb	Median	p25	p75	Mean ± SD	Min, Max	Reference
Serum TSH (mIU/L) ^a^	1.8	1.4	2.6	2.0 ± 0.9	0.6, 4.2	0.2–4.0
Serum fT_4_ (pmol/L)	16.0	14.5	17.2	15.9 ± 1.9	12.5, 20.1	11.0–23.0
Serum fT_3_ (pmol/L)	5.0	4.6	5.4	5.0 ± 0.5	4.3, 5.9	3.5–6.5
TPOAb (kU/L)	**n [%]**				**Min, Max**	
<34	13 [68]				<34,784	
≥34–100	4 [21]					<100
>100	2 [11]					

Differences between TSH, fT_4_, and fT_3_ were tested with the Mann–Whitney U test showing *p* = 0.051, *p* = 0.194 and *p* = 0.853, respectively. ^a^ One participant: elevated TSH and fT_4_ within reference range. TPOAb: peroxidase autoantibody; TSH: thyroid stimulating hormone; fT4: free thyroxine; fT3: free triiodothyronine (fT3).

**Table 5:** Frequency of habitual macroalgae consumption and procurement frequencies among macroalgae consumers in Norway (*n* = 44).

	Wholefood Macroalgae (*n* = 41)	Food Containing Macroalgae (*n* = 35)	Supplement Containing Macroalgae (*n* = 5)	All Product Categories (*n* = 44)	UIC, µg/L
**Frequency of seaweed consumption ^a^**	n [%] ^b^	n [%] ^b^	n [%] ^b^	n [%] ^b,c^	Median (p25–p75)
Daily	10 [23]	5 [11]	2 [5]	13 [30]	655 (253–2013)
4–6 times/week	8 [18]	5 [11]	0	10 [23]	2550 (564–6625)
1–3 times/week	16 [36]	7 [16]	1 [2]	15 [34]	1115 (203–2850)
Monthly	4 [9]	16 [36]	2 [5]	6 [14]	1525 (893–3438)
Other	3 [7]	2 [5]	0	0	-
**Procurement frequencies**	n [%] ^d^	n [%] ^d^	n [%] ^d^		
Personally harvested	17 [42]	7 [20]	0	-	-
Common food store (chain)	1 [2]	10 [29]	1 [20]	-	-
Common food store (not chain)	2 [5]	1 [3]	1 [20]	-	-
Online store	4 [10]	0	1 [20]	-	-
Health food store	2 [5]	0	0	-	-
Restaurant	0	2 [6]	0	-	-
Unspecified store	8 [20]	5 [14]	0	-	-
**Product declaration of iodine ^e^**					
Yes	9 [22]	5 [14]	2 [40]	-	-
No	21 [51]	25 [71]	2 [40]	-	-

^a^ Consumption frequencies: 41 used wholefood seaweed products, 35 used foods containing macroalgae (one missing), 5 used seaweed containing supplements. Multiple answers were allowed. ^b^ Percent of total number of participants (*n* = 44). ^c^ If different frequencies were given for different products, the most frequent were used. This might be underestimating the frequency of use, as different products may have been sued at different days. ^d^ Within macroalgae category: whole food macroalgae (*n* = 41); food containing macroalgae (*n* = 35); supplements containing macroalgae (*n* = 5).^e^ Eleven missing values for wholefood macroalgae, five missing values for food containing macroalgae, and one missing value for supplement containing macroalgae. Percentage is given within category, including missing.

**Table 6:** Habitual consumption (the last 4 weeks) of different macroalgae species reported among macroalgae consumers in Norway (*n* = 44).

Category	English	Latin	Habitual Consumption ^a^
			n [%] ^b^
**Brown algae**	Sugar kelp	*Saccharina latissima*	17 [39]
	Badderlocks, winged kelp	*Alaria esculenta*	16 [36]
	Bladderwrack	*Fucus vesiculosus*	5 [11]
	Sea spaghetti	*Himanthalia elongata*	5 [11]
	Wakame	*Undaria pinnatifida*	4 [9]
	Horned wrack	*Fucus serratus*	4 [9]
	Oarweed	*Laminaria digitata*	4 [9]
	Japanese kelp	*Laminaria japonica*/*Saccharina japonica*	2 [5]
	Rockweed, egg wrack	*Ascophyllum nodosum*	2 [5]
**Green algae**	Sea lettuce	*Ulva lactuca*	4 [9]
	Gut weed, mermaids’ hair	*Enteromorpha intestinalis*	1 [2]
**Red algae**	Dulse	*Palmaria palmata*	15 [34]
	Laver	*Porphyra purpurea*	8 [18]
	Wrack siphon weed	*Polysiphonia* (*vertebrata*) *lanosa*	1 [2]

^a^ Categorized from open-ended questions of whole food macroalgae products, multiple answers allowed; thus, several species may have been consumed for the participants under the specific species category. ^b^ Percent of total (*n* = 44).

## Supplementary Materials

The following are available online at https://www.mdpi.com/2072-6643/12/11/3483/s1, Table S1: Stability in UIC from the first to the second urine sample among Norwegian seaweed consumers (*n* = 44). Figure S1: Drop chart of UIC between 1st urine sample and 2nd urine sample, collected after 3–4 and 7–8-h after seaweed consumption. Figure S1a: normal-to-high UIC, Figure S1b: very high UIC, and Figure S1c: extremely high UIC. Table S2: Given reasons for including macroalgae in the diet among seaweed consumers in Norway (*n* = 44). Table S3: Overview of macroalgae used as part of meals in the diet (*n* = 44).
