# Supplementary material for: Iodine Status and Thyroid Function in a Group of Seaweed Consumers in Norway
Source: Nutrients. 2020 Nov 13;12(11):3483. doi: 10.3390/nu12113483 (PMC7697291; doi:10.3390/nu12113483)
Supplement: Supplementary file 1 [file nutrients-12-03483-s001.pdf]

Supplementary Materials:

Table S1. Stability in UIC from the first to the second urine sample among seaweed consumers in Norway (n=44).

	**UIC in 2nd urine sample**			**Correlation[b]**
**UIC in 1st urine sample**	Increase, n (%) [a]	Decrease, n (%) [a]	Stable, n (%) [a]	
< 1000 ug/L (n=22)	10 (45.5)	10 (45.5)	2 (9.1)	0.728**
> 1000 ug/L (n=22)	11 (50.0)	4 (18.2)	7 (31.8)	0.546*
All participants, n=44 (100 %)	21 (47.7)	14 (31.8)	9 (20.5)	0.886**

Increase and decrease were defined as a change larger than ± 10% from 1st to 2nd urine sample.

[a] Percent within categories. [b] Spearman's rho correlation coefficient. *Significant <0.05. **Significant <0.001.

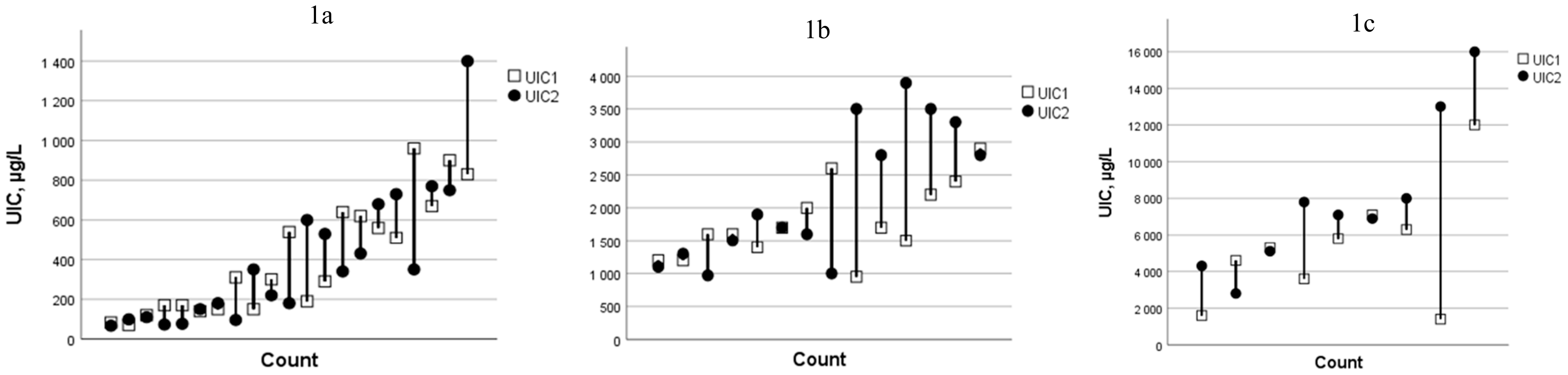

Figure S1 (a,b,c) Drop chart of UIC between 1st urine sample and 2nd urine sample, collected after 3-4 and 7-8 hour after seaweed consumption. **Figure S1a** normal to high UIC, **Figure S1b** very high UIC and **Figure S1c** extremely high UIC.

Table S2 Given reasons for including macroalgae in the diet among seaweed consumers in Norway (n=44)

Reasons for including macroalgae in the diet[a]	n [%][b]
Taste	29 [66]
Rich in nutrients	15 [34]
Sustainability	9 [21]
Positive health effects	8 [18]
Ensure enough iodine in the diet	7 [16]
Fun/ exciting	7 [16]
Healthy	6 [14]
A substitute for dairy or fish	4 [9]
Healing effect to cure illness	3 [7]
Reduce salt intake	3 [7]

[a] Categorized from open ended question, multiple answers allowed, two participants did not give a reason.
[b]Percent of total (n=44).

Table S3 Overview of how macroalgae are used as part of meals in the diet (n=44)

**Use of macroalgae as part of the diet**[a]	**n [%]**[b]
Unspecified	27 [61]
Seasoning/ spice	24 [55]
Sushi	8 [18]
As a snack/ side dish	8 [18]
In bread/ baked goods	5 [11]
As powder	3 [7]
Salad	3 [7]
Soup	3 [7]
Tablets	2 [5]
Pasta	2 [5]
Crisps/ chips	2 [5]
Wok	1 [2]
In smoothie	1 [2]

[a] Categorized from open ended question, multiple answers allowed, two participants did not give a reason. [b]Number of times mentioned. Participants could have mentioned one category of usage several times (e.g. one time for whole food and one time for foods containing macroalgae).
